# Supplementary material for: Mechanisms governing the pioneering and redistribution capabilities of the non-classical pioneer PU.1
Source: Nat Commun. 2020 Jan 21;11:402. doi: 10.1038/s41467-019-13960-2 (PMC6972792; doi:10.1038/s41467-019-13960-2)
Supplement: Supplementary file 7 — Source data [file 41467_2019_13960_MOESM7_ESM.zip › Source_Data/FigureS5/FigureS5A_MotifsCommonPeaks/homerResults/motif54.similar.html]

motif54

## Information for motif54

G
A
C
T
A
G
C
T
G
A
T
C
A
G
C
T
A
G
T
C
C
T
G
A
G
A
T
C
T
C
A
G
T
C
G
A
A
C
T
G
G
T
C
A
C
G
A
T
A
G
T
C
  
Reverse Opposite:  

T
C
A
G
G
C
T
A
C
A
G
T
T
G
A
C
A
G
C
T
A
G
T
C
C
T
A
G
G
A
C
T
C
T
A
G
C
T
G
A
C
T
A
G
T
C
G
A
C
T
G
A
  

|  |  |
| --- | --- |
| p-value: | 1e-15 |
| log p-value: | -3.601e+01 |
| Information Content per bp: | 1.665 |
| Number of Target Sequences with motif | 90.0 |
| Percentage of Target Sequences with motif | 1.01% |
| Number of Background Sequences with motif | 115.3 |
| Percentage of Background Sequences with motif | 0.30% |
| Average Position of motif in Targets | 103.8 +/- 64.2bp |
| Average Position of motif in Background | 107.8 +/- 63.0bp |
| Strand Bias (log2 ratio + to - strand density) | -0.1 |
| Multiplicity (# of sites on avg that occur together) | 1.05 |
| Motif File: | file (matrix) reverse opposite |

### Similar de novo motifs found

|  |  |  |  |  |  |  |  |
| --- | --- | --- | --- | --- | --- | --- | --- |
| Rank | Match Score | Redundant Motif | P-value | log P-value | % of Targets | % of Background | Motif file |
| 1 | 0.865 | G A C T A G T C A G C T G T A C A C T G A G T C A C T G G T C A C A T G G T C A G A T C A G T C | 1e-15 | -35.272028 | 0.56% | 0.09% | motif file (matrix) |
| 2 | 0.935 | T G C A A G C T G T A C A G C T A T G C C T A G G A T C T A C G T C G A C A T G C T G A | 1e-15 | -34.726152 | 1.46% | 0.58% | motif file (matrix) |
| 3 | 0.778 | C G A T G T A C A C G T A G T C A C T G A G T C A C T G C G T A | 1e-13 | -29.969995 | 0.71% | 0.19% | motif file (matrix) |
| 4 | 0.870 | A C G T T A G C A G C T A G T C C T G A G C A T C T A G C T G A T C A G C G T A C T G A A G T C | 1e-12 | -27.981665 | 0.89% | 0.30% | motif file (matrix) |
